# Neural circuits for peristaltic wave propagation in crawling *Drosophila* larvae: analysis and modeling

**DOI:** 10.3389/fncom.2013.00024

**Published:** 2013-04-04

**Authors:** Julijana Gjorgjieva, Jimena Berni, Jan Felix Evers, Stephen J. Eglen

**Affiliations:** ^1^Department of Applied Mathematics and Theoretical Physics, Centre for Mathematical Sciences, University of CambridgeCambridge, UK; ^2^Department of Zoology, University of CambridgeCambridge, UK

**Keywords:** *Drosophila*, CPG, sensory feedback, synchronous, wave propagation

## Abstract

*Drosophila* larvae crawl by peristaltic waves of muscle contractions, which propagate along the animal body and involve the simultaneous contraction of the left and right side of each segment. Coordinated propagation of contraction does not require sensory input, suggesting that movement is generated by a central pattern generator (CPG). We characterized crawling behavior of newly hatched *Drosophila* larvae by quantifying timing and duration of segmental boundary contractions. We developed a CPG network model that recapitulates these patterns based on segmentally repeated units of excitatory and inhibitory (EI) neuronal populations coupled with immediate neighboring segments. A single network with symmetric coupling between neighboring segments succeeded in generating both forward and backward propagation of activity. The CPG network was robust to changes in amplitude and variability of connectivity strength. Introducing sensory feedback via “stretch-sensitive” neurons improved wave propagation properties such as speed of propagation and segmental contraction duration as observed experimentally. Sensory feedback also restored propagating activity patterns when an inappropriately tuned CPG network failed to generate waves. Finally, in a two-sided CPG model we demonstrated that two types of connectivity could synchronize the activity of two independent networks: connections from excitatory neurons on one side to excitatory contralateral neurons (E to E), and connections from inhibitory neurons on one side to excitatory contralateral neurons (I to E). To our knowledge, such I to E connectivity has not yet been found in any experimental system; however, it provides the most robust mechanism to synchronize activity between contralateral CPGs in our model. Our model provides a general framework for studying the conditions under which a single locally coupled network generates bilaterally synchronized and longitudinally propagating waves in either direction.

## Introduction

Central pattern generator (CPG) circuits are autonomous groups of neurons, or neural networks, that produce patterned, rhythmic neural output in the absence of sensory or descending inputs that carry specific timing information (Marder and Bucher, 2001). These networks underlie the production of most rhythmic motor patterns such as breathing, walking, and swimming (Marder and Calabrese, 1996; Grillner, 1999, 2003). CPG circuits have been studied in several vertebrate and invertebrate systems (Grillner, 2003, 2006; Marder et al., 2005), leading to a detailed understanding of the cellular and circuit mechanisms that generate rhythmic motor patterns. We have studied the locomotor generating circuit in *Drosophila* larvae. *Drosophila* larvae exhibit highly stereotyped locomotor behavior characterized by the synchronous contraction of muscles on the left and the right side of the body. During forward crawling, peristaltic waves of muscle contractions travel from posterior to anterior abdominal segments of the larva, while the reverse occurs during backward crawling (Fox et al., 2006; Dixit et al., 2008; Lahiri et al., 2011; Berni et al., 2012; Heckscher et al., 2012). This rhythmic movement can be generated independently of sensory feedback and descending input from the brain, suggesting the existence of a CPG network that underlies this behavior in the thoracic and abdominal segments (Suster and Bate, 2002; Hughes and Thomas, 2007; Berni et al., 2012). This is in contrast to models of the nematode *C. elegans* where sensory feedback from the motor circuit has been shown to be the critical element for rhythm generation (Wen et al., 2012).

In some vertebrates, such as lamprey and *Xenopus*, the locomotor CPG that generates swimming movements is organized into two reciprocally inhibitory “half centers” located on each side of the spinal cord (Arshavsky et al., 1993). These half centers are connected by commissural interneurons, thus producing alternating left-right activity in the two half centers (Soffe et al., 1984; Grillner, 1985; Arshavsky et al., 1993). The left-right synchronous muscle contractions during crawling in *Drosophila* larvae stand in contrast to the left-right alternating contractions during swimming in lamprey and tadpole (or dorso-ventral in leech). Therefore, existing CPG models of these and other experimental systems generating alternating activity patterns (Cang and Friesen, 2002; Grillner, 2003, 2006; Marder et al., 2005; Bryden and Cohen, 2008; Mullins et al., 2011) are inadequate for modeling peristaltic wave propagation as observed in *Drosophila* crawling.

Here we first quantified the behavior of freely crawling *Drosophila* larvae by calculating the duration of contraction of abdominal segments and the speed of propagation from one segment onto the next. Based on these data we developed a new CPG model to study locomotor wave pattern generation. Our model is an abstraction of neural circuits that might generate larval crawling and captures the observed coordination at the segmental level. In the model, the activity in each abdominal segment is represented with a unit consisting of excitatory and inhibitory (EI) neuronal populations. These units are then coupled to allow for the propagation of activity. We showed that a minimal fully symmetric network architecture consisting of nearest-neighbor excitatory and inhibitory connections can generate unidirectional propagating waves qualitatively matching experimental observations of propagating segmental contractions. The same network produced both forward and backward waves evoked by the activation of the most posterior, or most anterior segment, respectively. Therefore, *Drosophila* larvae might not require independent networks for forward and backward crawling, but could use a symmetric layout to support forward and backward locomotion. We found that wave generation is more sensitive to changes in excitatory than inhibitory intersegmental connectivity. Whether such different requirements for precise tuning might be reflected in biology by higher variability in inhibitory than excitatory connectivity will need to be addressed experimentally. Incorporating sensory feedback based on “stretch-sensitive” neurons (Suster and Bate, 2002; Fox et al., 2006; Hughes and Thomas, 2007) produced waves where segmental contraction duration and speed of propagation were close to experimental observations. Sensory feedback also improved network robustness; it successfully restored propagating waves in networks lacking sensory feedback that did not generate waves due to inappropriate parameter settings. Finally, we considered two networks which can independently generate propagating activity, and determined how to connect them while ensuring that their activity was synchronous. Thus, our modeling proposes plausible ways to connect left and right networks to obtain synchronous propagation of activity in both left and right hemisegments, different from the cross-inhibitory connections required for producing alternating activity in other animals.

## Materials and methods

### Crawling behavior analysis

**Figure 1:**
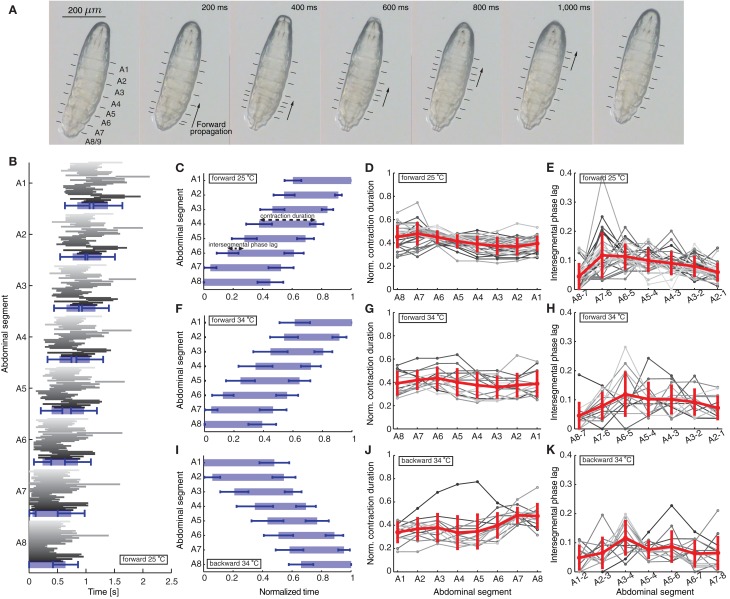
**Quantification of peristaltic waves of contraction during forward and backward crawling in a first instar *Drosophila* larva. (A)** One wave of peristaltic crawling in first instar larva is illustrated through snapshots taken every 200 ms. The individual panels show the forward propagation of segmental contractions. The segments are labeled from posterior to anterior as A8/9 through A1 (see also Figure 2A). The arrows illustrate the simultaneous contraction of several neighboring segments during a wave, which propagates from posterior (segment A8/9) to anterior end (segment A1) of the larva. The lines in each panel mark the position of the dentical belts approximating segmental boundaries. **(B)** Time of contraction of each segment for the 35 forward waves (shown in different shades of gray) recorded at 25°C. The 35 waves recorded in 12 animals (not all consecutive) show a great variability among different animals and different waves in the same animal. The blue rectangles denote averages, while the blue bars standard deviations of the start and end time of contraction of each segment. Only forward waves were measured where the larvae did not perform left/right turning or exploratory head extensions. The larvae did not crawl backward spontaneously. **(C)** Mean ± SD of the segmental contraction durations of each segment for the same 35 waves in **(B)**. Each segmental contraction duration was normalized by the total wave duration of the corresponding wave, and then averaged. We also illustrate the normalized contraction duration and the intersegmental phase lag quantified in **(D)** and **(E)**. **(D)** Normalized contraction duration for each abdominal segment for the 35 forward waves in **(B)** in different shades of gray. Mean ± SD is shown in red. **(E)** Intersegmental phase lag for the 35 forward waves in **(B)** in different shades of gray. Mean ± SD is shown in red. **(F,I)** Mean ± SD of the normalized segmental contraction durations of each segment for 15 forward waves **(F)** and 14 backward waves **(I)** recorded at 34°C. **(G,J)** Normalized contraction duration for each abdominal segment for the 15 forward waves **(G)** and 14 backward waves **(J)** recorded at 34°C in different shades of gray. Mean ± SD is shown in red. **(H,K)** Intersegmental phase lag for the 15 forward waves **(H)** and 14 backward waves **(K)** recorded at 34°C in different shades of gray. Mean ± SD is shown in red.

Newly hatched first instar *Drosophila* larvae (wild type strain Oregon-R) were placed on a 5 cm Petri dish coated with 0.9% agarose. Thirty second movies were captured at 30 frames per second with a JVC TKC1380 camera adapted on a Leica M420 microscope. The contraction of one particular segment was defined as the time between the first movement of the posterior denticle band and the last movement of the anterior denticle band that define its boundaries (see Figure 1). The timing of contraction was quantified with the open source software VCode 1.2.1 (http://social.cs.uiuc.edu/projects/vcode.html) (Hagedorn et al., 2008).

We first quantified a total of 35 forward waves in 12 animals recorded at 25°C (Figures 1B–E). These waves were not all consecutive, because a requirement for quantification was that the entire animal was in view under the microscope during each wave. In this paper we focused only on characterizing and modeling waves propagated by the 8 abdominal segments. In between these waves, the larvae frequently move their head and thoracic segments, which we did not consider. However, we found no significant difference between the time of propagation of the first peristaltic wave following a pause, compared to other consecutive waves (first wave 1180 ± 29 ms and following waves 1235 ± 16 ms; *t*-test: *t* = 1.521, df = 217, *p* = 0.1297). Since we quantified multiple consecutive waves in several animals, we also calculated the duty cycle (although we did not compare it to the model). Using the formula *D* = (time of wave propagation)/(stride period), where stride period denotes the total time of wave propagation and the time between waves, we found that *D* = 0.83 ± 0.22 (mean ± SD). We also quantified 15 forward and 14 backward waves in 10 animals in arenas where the temperature was raised to 34°C imposing the same requirements (Figures 1F–K). All quantities in the text have been reported as mean ± SD.

### A wilson-cowan model of a single segment

The activity in each segment was modeled with a Wilson–Cowan unit (Wilson and Cowan, 1972) consisting of two neuronal populations, excitatory (*E*) and inhibitory (*I*). These two populations represent the joint activity of all central neurons in the CPG circuit for crawling. The differential equations for the time-dependent variation of averaged excitatory and inhibitory neuronal activities were

(1) $$\tau_{E}\dot{E}=-E+(k_{E}-E)G_{E}(aE+cI+P_{\text{ext}})$$

(2) $$\tau_{I}\dot{I}=-I+(k_{I}-I)G_{I}(eE+fI),$$

where the functions *G*_*E*_, *G*_*I*_, and *G*_*S*_ represent sigmoidal response functions of the excitatory, inhibitory, and sensory neuronal populations given by

(3) $$G(x)=\frac{1}{1+\exp\left[-\lambda (x-\theta )\right]}-\frac{1}{1+\exp(\lambda \theta )},$$

λ represents the maximum slope of the sigmoid (or if *G* represents an activation function, it denotes the speed of activation) and θ represents the location of the maximum slope (or the threshold for activation). The terms *k*_*E*_ and *k*_*I*_ denote the maxima of the response functions for the excitatory and inhibitory populations, *k*_*E*_ = 0.9945 and *k*_*I*_ = 0.9994, obtained for λ_*E*_ = 1.3 and λ_*I*_ = 2 (Wilson and Cowan, 1972). The time constants τ_*E*_ and τ_*I*_ denote the decay of the excitatory and inhibitory activities after stimulation, and determine the timescale of activity in the network. All parameters were dimensionless and time in the model was measured in arbitrary time units (t.u.). The time constants for the dynamics, τ_*E*_ and τ_*I*_, were chosen such that one time unit in the model corresponded to roughly one second of segmental contraction duration in our experiments. The variability of the waves in our data suggest that *Drosophila* larvae crawl at different speeds which depend on other factors excluded from our model (Figure 1B). The normalized segment activity duration and intersegmental phase lag (defined below) were independent of the exact choice of these time constants, and were therefore used throughout the paper to allow for comparison between the waves recorded experimentally and model waves.

The connectivity coefficients *a* (or *e*) and *c* (or *f*) represent the average number of excitatory and inhibitory synapses per cell in the excitatory (or inhibitory) population, respectively. The time-varying function *P*_ext_(*t*) denoted the external input applied to the excitatory population. The system of Equations (1)–(2) has been previously analyzed (Wilson and Cowan, 1972; Borisyuk and Krilov, 1992). Depending on the parameters in the system, there can be multiple equilibria with different stability properties; but here we used the values from Wilson and Cowan (1972) (see Table 1), for which the system has a single unstable fixed point and a stable limit cycle in response to constant stimulation *P*_ext_. This means that applying constant input to such an isolated unit, the activity of the excitatory and inhibitory populations oscillates (Wilson and Cowan, 1972; Borisyuk and Krilov, 1992; Borisyuk et al., 1995). However, when multiple segments are connected as in our network (see below), due to the presence of inhibition from neighboring segments, oscillations are suppressed and instead waves can be propagated across the segments.

**Table 1:** **Network parameters for wave propagation**.

***a***	***b***	***c***	***d***	***e***	***f***	τ_***E***_	τ_***I***_	τ_***S***_	***P*_ext_**	***b*_*E*_**	***b*_*I*_**	***b*_*S*_**	θ_***E***_	θ_***I***_	θ_***S***_
16	20	−12	−20	15	−3	0.5	0.5	0.5	1.7	1.3	2	1.3	4	3.7	2

*Default parameter values for the model simulations*.

### A network model for wave propagation with interconnected segments

We modeled propagating waves only in the eight abdominal segments, excluding any head and thoracic segment movements typically performed by the larvae between abdominal segment waves. Therefore, our model could not capture the data about duty cycles as this data incorporates such additional movements of the head and thoracic segments.

**Figure 2:**
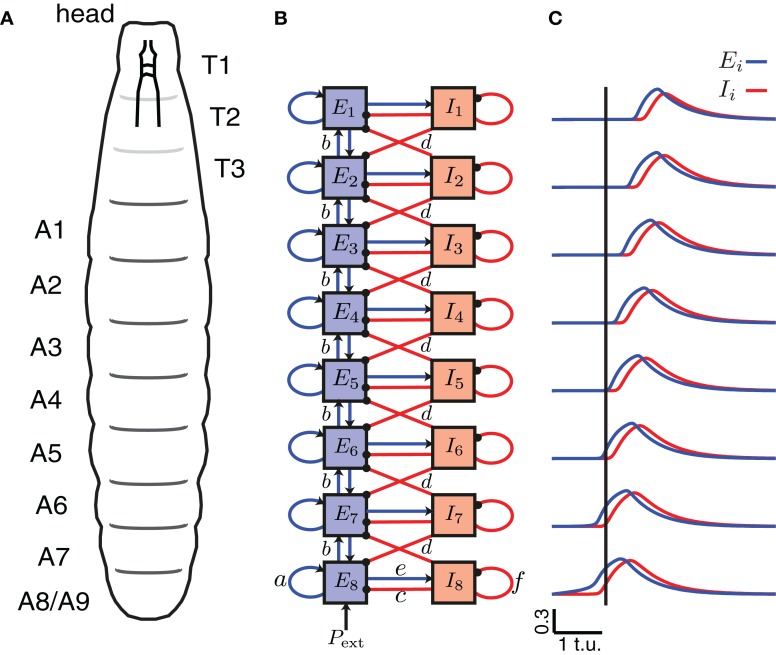
**A CPG network model of the segmentally repeated body of a *Drosophila* larva. (A)** A schematic of the body of a *Drosophila* larva. T1–T3 denote the thoracic segments (not analyzed/modeled), and A1–A8 the abdominal segments. **(B)** A network model for peristaltic wave propagation with eight interconnected segments, each consisting of an excitatory (*E*) and an inhibitory (*I*) neuronal population. Connections *a*, *c*, *e*, and *f* are shown only for segment A8, but are found in each segment. The segments are connected with nearest-neighbor excitatory, *b* (blue, arrows), and inhibitory, *d* (red, dots), connections. Forward waves are initiated by providing external input *P*_ext_ into the excitatory population of segment A8. **(C)** The excitatory activity for each of the eight segments is shown in blue, and the inhibitory activity in red during a forward wave as a function of time. A vertical line is drawn where *E*_8_ exceeds threshold to highlight that the wave progresses from posterior to anterior.

To model wave propagation with segmental activity patterns as those observed during *Drosophila* crawling, we coupled eight Wilson–Cowan EI units described above representing the eight repeated abdominal segments of the *Drosophila* body (A8–A1) (Figures 2A,B). The activity of the excitatory and inhibitory populations in each segment, *j*, was represented with the averaged firing rates *E*_*j*_(*t*) and *I*_*j*_(*t*), respectively. Between segments, nearest-neighbor connections of two types were used: bidirectional excitatory connections (*b*) between the excitatory populations of neighboring segments leading to a propagation of activity along the chain of segments, and inhibitory connections (*d*) from the inhibitory population in one segment to the neighboring (anterior and posterior) excitatory populations (Figure 2B) to terminate activity in the previously active segment and ensure unidirectional wave propagation. This network was equipped with symmetric bidirectional connectivity such that forward and backward propagating waves were generated with the same properties. We list typical values for these parameters of the model in Table 1, which were determined from the exploration in Figure 5.

The equations for the averaged firing rates with *i* = 1, …, 8 can be written as:

(4) $$\tau_{E}{\dot{E}}_{i}=-E_{i}+(k_{E}-E_{i})G_{E}(bE_{i-1}+aE_{i}+bE_{i+1}-dI_{i-1}-eI_{i}-dI_{i+1})$$

(5) $$\tau_{I}{\dot{I}}_{i}=-I_{i}+(k_{I}-I_{i})G_{I}(cE_{i}-fI_{i})$$

where we note that the terms in the equations for *i* = 1 (*i* = 8) containing *i* − 1 (*i* + 1) drop out, because the end segments (A1 and A8) received excitation and inhibition only from one neighboring segment.

Other alternative architectures might be similarly suited to generate propagating waves. Here we strived to use a network of minimal connection complexity to reveal the general network properties that support crawling behavior as described in this paper.

### Incorporating sensory feedback in the model

The equations of the model with sensory feedback are an extension of the Equations (1)–(5), with additional input from the sensory populations for *i* = 1, …, 8

(6) $$\tau_{E}{\dot{E}}_{i}=-E_{i}+(k_{E}-E_{i})G_{E}(bE_{i-1}+aE_{i}+bE_{i+1}-dI_{i-1}-eI_{i}-dI_{i+1}+\beta S_{i})$$

(7) $$\tau_{I}{\dot{I}}_{i}=-I_{i}+(k_{I}-I_{i})G_{I}(cE_{i}-fI_{i}+\gamma S_{i})$$

where the dynamics for the sensory populations are

(8) $$\begin{array}{l} \tau_{S}{\dot{S}}_{1}=-S_{1}+G_{S}(\alpha {\left[E_{2}-E_{1}\right]}_{+})\\ \;\ldots \ldots \end{array}$$

(9) $$\begin{array}{l} \tau_{S}{\dot{S}}_{1}=-S_{i}+G_{S}(\alpha {\left[E_{i+1}-E_{i}\right]}_{+}+\alpha {\left[E_{i-1}-E_{i}\right]}_{+})\\ \;\ldots \ldots \end{array}$$

(10) $$\tau_{S}{\dot{S}}_{8}=-S_{8}+G_{S}(\alpha {\left[E_{7}-E_{8}\right]}_{+}),$$

for *i* = 2, …, 7. Here, [*x*]_+_ = max(*x*, 0). For the activation function of the sensory neuronal populations, we used a sigmoid *G*_*S*_ with the same slope as the excitatory population, *b*_*s*_ = *b*_*e*_ = 1.3, but a threshold for activation smaller than that for the excitatory population θ_*s*_ = 2 < θ_*e*_ such that sensory activity increases before excitatory activity. The time constant τ_*S*_ was equal to τ_*E*_ because wave properties were reported normalized to wave duration.

### Characterization of waves in the model

**Figure 3:**
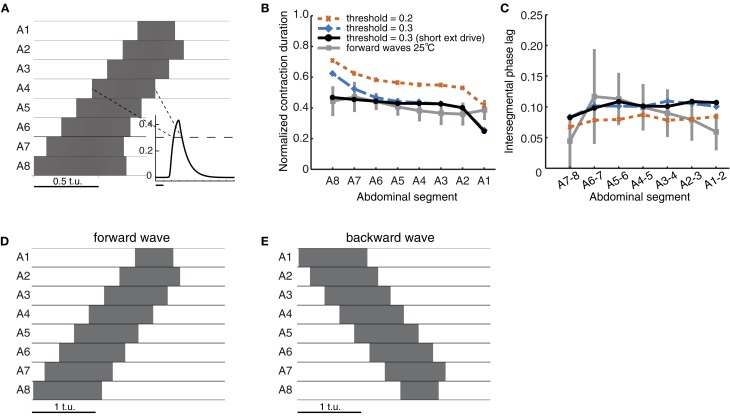
**Quantification of forward peristaltic waves in the CPG network model. (A)** An example of suprathreshold excitatory activity (θ_*C*_ = 0.3, dashed line in inset) of each segment. Time is measured in arbitrary time units (t.u.). External drive *P*_ext_ = 1.7 was applied to *E*_8_ for 2 t.u. **(B)** Duration of suprathreshold excitatory activity (corresponding to contraction) for each segment normalized by the wave duration for waves generated with different thresholds. For comparison, we have also plotted the experimental data from forward waves at 25°C (Figure 1D). **(C)** Intersegmental phase lag for the same waves in **(B)**. We also show the experimental data from forward waves at 25°C (Figure 1E). **(D)** Same as **(A)** except that the external drive was applied for 1 t.u. **(E)** To generate backward waves, similar parameter values were used as in **(A)**, but with external drive applied to E1 (not E8).

A “contraction” in the model was defined as suprathreshold activity (activity above a threshold θ_*C*_) of the excitatory population (Figure 3A). Therefore, we compared the duration of suprathreshold excitatory activity in the model to the duration of segmental contractions in experimental data, and threshold crossing in the model to onset of contraction in the data (Figure 1B). The *normalized contraction duration* was defined as the contraction duration of each segment (from the onset until the offset of contraction in that segment) divided by the total duration of the wave (time of contraction offset in segment A1 minus time of contraction onset in segment A8). The *intersegmental phase lag* was defined as the time from the start of contraction of one segment to the start of contraction of the next neighboring anterior segment, divided by the wave duration.

### Robustness simulations

Forward waves generated by the model (Figure 5) were analyzed only if the excitatory activity of a given segment increased above threshold after the excitatory activity of the posterior neighboring segment, and then decreased below threshold after posterior excitatory activity—as should occur during forward wave propagation. For waves that did not meet these criteria, and when waves could not be initiated, the value of 0 was used (Figures 5A–C). A wave in Figure 6 was considered successful when the external drive *P*_ext_ = 1.7 applied for a duration of 1.2 t.u. initiated a single wave only, and also when all the segments were activated above threshold in the correct order (posterior before anterior), and then deactivated in the same order, except for the last segment A1 which always deactivated before A2 in the simulations without sensory feedback.

### Two-sided model

To initiate a wave in the two-sided model (Figure 9), an external input was applied to the two excitatory populations in segment A8, with a small difference in the strength to each side (e.g., *P*_ext_ = 1.7 to the left side and *P*_ext_ = 1.72 to the right side) to avoid instantaneous synchronization due to the deterministic nature of the model. A simulation was performed for each parameter choice, and after disregarding transient activity, we computed the difference in timing between the threshold crossing of *E*^left^_*j*_ and *E*^right^_*j*_, averaged across all segments *j* = 1, …, 8. Waves where the difference between excitatory activity on the left and the right side was longer than 0.1 t.u. were discarded (Figure 10).

## Results

### Characterizing crawling patterns in *Drosophila* larvae

*Drosophila* larvae can crawl at different speeds, but during undisturbed spontaneous crawling the patterns are highly stereotypical (Figure 1) (Suster, 2000; Fox et al., 2006; Lahiri et al., 2011; Berni et al., 2012; Heckscher et al., 2012). During forward crawling, a wave is typically initiated by the simultaneous contraction of the most posterior segments, A8/9. As the contraction propagates anteriorly, each of the remaining abdominal segments (A7–A1) is transiently lifted from the substrate, propelled forward, and attached again to the substrate through a belt of cuticular denticles which serve as anchorage points (Crisp et al., 2008; Dixit et al., 2008; Berni et al., 2012) (Figure 1A). Segments anterior to the abdomen (head and thorax) move differently (Dixit et al., 2008; Heckscher et al., 2012).

We have therefore focused on the propagation of contraction waves in the abdominal segments, complementing experimental observations with a modeling study. Figure 1 shows the propagation of segmental contractions during a peristaltic wave of a freely crawling newly hatched *Drosophila* larva. The larva is shown from the bottom with horizontal lines denoting the segmental boundaries. We recorded the times when each segment started and stopped contracting for 35 waves in 12 different animals (Figure 1B) (see Materials and Methods for how we selected these waves). Consistent with previously reported data by Heckscher et al. (2012), the average wave duration was 1.39 ± 0.25 s for forward crawling. The waves within the same animal and between different animals were variable (Figure 1B). Therefore, we reported the average duration of contraction for the different segments normalized by the total wave duration (Figure 1C). Each segment contracted 0.415 ± 0.076 of the time during which the wave propagated along the body.

We also quantified the intersegmental phase lag between contractions (time between the start of contraction of two neighboring segments, see Materials and Methods). The intersegmental phase lag, shown in Figure 1D for neighboring segment pairs, was 0.087 ± 0.050 of the total wave duration for all pairs. This intersegmental phase lag could be interpreted as inversely proportional to the normalized propagation speed of a wave. The contraction propagation is fastest through the most posterior pair of segments (A8 and A7): the phase lag was significantly faster through A8 and A7 than through the next fastest segments (A2 and A1) (*p* = 0.03; Mann–Whitney two-tailed test). This is consistent with a model where a certain threshold level needs to be overcome for a wave to start.

Newly hatched first instar larvae generally crawl forward (99.8%), but can briefly move backward in response to sensory stimuli to the head (Kernan et al., 1996), increased temperature (Berni et al., 2012) or certain constrained conditions (Heckscher et al., 2012). During backward movement, the wave of contractions is reversed and passes from anterior to posterior. Previous studies have quantified backward waves for larvae in linear channels, and found that larvae crawl backward at a lower speed than forward (Heckscher et al., 2012). To examine the activity pattern of backward crawling in unrestrained conditions, we recorded 14 backward waves at 34°C when *Drosophila* larvae crawl backward (Figure 1I). We also quantified 15 forward waves at 34°C (Figure 1F), allowing us to compare the two types of waves under the same condition. Examining the normalized contraction duration and intersegmental phase lag for these waves (Figures 1G,H,J,K) revealed that although at 34°C waves were more variable, the wave properties, both normalized contraction duration and intersegmental phase lag, were similar to the forward waves recorded at 25°C.

### Characterizing wave propagation in the model

Next, we proposed and analyzed a minimal network architecture that might underlie the generation of peristaltic wave propagation as observed during crawling in *Drosophila* larvae. We first concentrated on a model that captured the segmental activity of a whole segment and subsequently studied left/right synchronization. The following criteria were used to design the CPG model:

1. The network output should be rhythmic [as required for a CPG (Marder and Bucher, 2001)] such that external drive initiating the waves should not deliver any timing information. The timing properties of wave propagation should be governed purely by the dynamics of segmental activity in the network.
2. The network should produce a propagating wave from one end to the other, with duration and timing of activity in neighboring segments matching experimental recordings of crawling in *Drosophila* larvae (Figures 1C–E).
3. Network connectivity should be symmetric such that a single network supports wave propagation in both directions, from posterior to anterior (as during forward crawling), and from anterior to posterior (as during backward crawling) (Figures 1I–K).
4. The network should produce propagating waves without sensory feedback characteristic of a CPG-driven network (Suster and Bate, 2002; Crisp et al., 2008).
5. Sensory feedback to the CPG network should modulate its output as previously shown in experimental studies (Hughes and Thomas, 2007).

The model architecture is based on coupled neuronal populations, excitatory and inhibitory, which represent the CPG circuit underlying crawling. Thus, our model captures the gross crawling behavior observed at the segment level, and does not explicitly incorporate motorneuron or muscle dynamics. There is little experimental evidence on the specific identity, anatomy, and synaptic connectivity of central neurons that generate the basic crawling rhythm; thus we aim to explore minimal models that can generate the above behaviors and elucidate general principles behind connectivity of the motor networks for larval crawling. Our model aims at providing constraints on future modeling studies, but also at suggesting testable predictions for future biological experiments.

A schematic of the larval body is illustrated in Figure 2A showing its segmented organization. We modeled only the abdominal segments (A8–A1), using a circuit consisting of 8 coupled excitatory and inhibitory population units (Figure 2B). Previous work has explored how varying the intrasegmental connectivity parameters (*a*, *e*, *c*, and *f*, Table 1) in a single segment (EI unit) affects the activity of the E and I populations in that unit (Wilson and Cowan, 1972; Borisyuk and Krilov, 1992); here we study the effects of variability in the intersegmental connectivity (*b* and *d*, Table 1).

Since peristaltic waves in *Drosophila* larvae start crawling forward by the contraction of the most posterior segment, we assumed that activation of A8 elicits the beginning of a forward wave. To initiate a single forward wave in the CPG network (Figure 2B), a constant, external input *P*_ext_ of fixed duration was applied to *E*_8_ (Figures 3A,D), while to initiate a single backward wave, *P*_ext_ was applied to *E*_1_ (Figure 3E). No external input was provided to the inhibitory populations, nor to the other excitatory populations in the model. Once the excitatory population in the posterior segment, *E*_8_, was driven beyond the threshold for activation, activity propagated to the other excitatory populations (Figure 2C).

Excitatory activity in the model was evaluated while it exceeded a certain threshold (θ_*C*_; see Materials and Methods). We compared the timing of such *suprathreshold* excitatory activity in the model to the timing of segmental contractions in the data. We tested thresholds θ_*C*_ ın [0.2, 0.3] (activity was bounded in [0,0.5]) (Figures 3B,C). The normalized segment contraction duration generated with the model matched that in the experimental data (Figure 3B), except for one notable difference. While the normalized contraction duration of all segments in the experiments was similar (Figure 1C), in the model, the posterior segment *E*_8_ was active for longest, and the anterior segment *E*_1_ for shortest (Figure 3B). This bias originates in the design of the model, namely, the two end segments in the network receive inputs of different strength: during forward wave propagation, *E*_8_ receives external input *P*_ext_ and also excitatory input from its neighbor *E*_7_; *E*_1_, however, received excitatory input only from its neighbor *E*_2_ and no external input. We later show that this difference can be overcome by adding sensory input to the model (see Figure 7). The difference can also be overcome by reducing the duration of *P*_ext_, which shortens the duration of suprathreshold activity in *E*_8_ (Figure 3D). Interestingly, a qualitatively similar difference has also been observed in experiments where activity from the segmental nerve of posterior and anterior segments was recorded (Fox et al., 2006). In particular, the recorded bursts tended to be longer in segments where the waves originated, so in the case of forward waves, longer bursts were observed in posterior segments. In this work, however, the segmental activity was recorded for pairs of segments only (Fox et al., 2006). It would be interesting to compare our simulated waves to recordings of all segments. In addition to the normalized segment contraction duration, we also examined the intersegmental phase lag for the model waves. Figure 3C shows that the intersegmental phase lag was in the same range as for the experimentally observed waves.

For most models we detected suprathrehsold activity using a threshold of θ_*C*_ = 0.3. Decreasing this threshold increased the duration of suprathreshold activity and, thus, the normalized segment duration (Figure 3B). Decreasing the threshold also increased the speed of propagation (by decreasing the time between activation of neighboring segments) relative to the overall wave duration (Figure 3C). Backward waves had the same normalized contraction duration and intersegmental phase lags (Figures 3D,E). This is due to the bidirectionally-symmetric connections between neighboring segments in this model and because of the way backward waves were elicited by applying the external drive *P*_ext_ to the most anterior segment A1 instead of A8.

### Continuous external drive can evoke multiple waves

**Figure 4:**
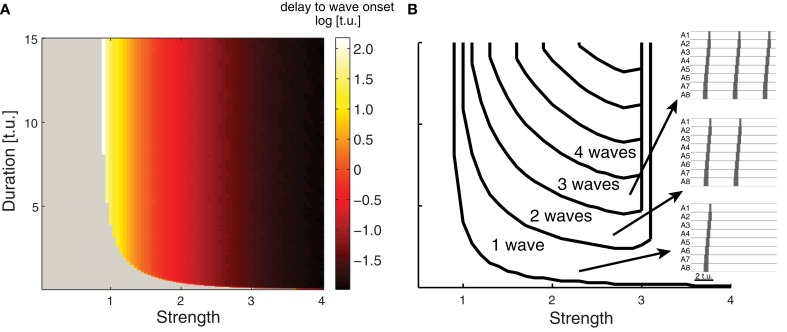
**Evoking multiple waves in the CPG network model. (A)** The time delay (indicated by the color) between applying external input and wave onset for a range of external inputs whose strength and duration are plotted on the abscissa and ordinate axes. Gray indicates that no wave was initiated, which occurs for weak and short external drive. **(B)** Number of waves generated for different external inputs. As the strength and duration of external input increases, more waves are generated (examples on right) with properties as in Figure 3. When the strength of the external input is larger than 3.1 (regardless of duration) a single wave is initiated; the first segment in this wave is active for a longer period of time compared to the other segments (duration depends on the external drive duration).

Network activity gradually rises upon the onset of an external stimulus *P*_ext_ due to several factors including network connectivity and the intrinsic dynamics of the *E* and *I* populations in each EI unit. Figure 4A shows the time required for wave initiation as the duration and intensity of *P*_ext_ is varied. This agrees with the original analysis performed for a single EI unit by Wilson and Cowan (1972), who derive the intensity and duration of a rectangular impulse which is sufficient for the excitatory population to become self-exciting and transition from the quiet to the excited state. In our model of 8 connected segments, a single peristaltic wave is generated for short and weak external drive duration, and the network returns to a quiet state. If the network receives an external input of intermediate strength, multiple waves can be generated for as long as the stimulus is applied (Figure 4B). This demonstrates that multiple waves with appropriate duration can be generated by suitably adjusting the external input, and might explain how continuous *straight* crawling is maintained in *Drosophila* larvae. However, *Drosophila* larvae often pause and turn (Kernan et al., 1996; Berni et al., 2012), performing movements coordinated by the head and the thoracic segments which we did not model. As a result, our findings cannot account for waves involving turning and decision making which occur during spontaneous forward crawling. The continuous rhythmic output that we observe in Figure 4 is generated by the network; external input is required for the waves to occur, but delivers no timing information for the individual waves. For instance, in the presence of constant external input, upon the completion of each wave, a new wave will begin with the activation of the the posterior segment A8 once the external input overcomes network inhibition to *E*_8_ elicited by the previous wave. If the external drive is stronger than 3.1, regardless of duration a single wave is generated, but the first segment in this wave remains continuously active for a long period of time depending on the duration of the drive. In the remainder of the paper we generated and quantified single waves by applying external drive with appropriate strength (see Table 1) and duration.

### Intersegmental connectivity strength modifies speed and phase lag of wave propagation

How does wave generation and propagation depend on the connectivity of the proposed network? Since the connectivity of a single EI unit used to model one segment has already been studied (Wilson and Cowan, 1972; Borisyuk et al., 1995), we examined the strength of intersegmental connections (*b* and *d* in Figure 2B). To understand the balance between excitatory and inhibitory connectivity, we performed two studies:

1. We analyzed the effect of varying the strength of excitatory *b* and inhibitory *d* intersegmental connections. All excitatory or inhibitory connections were varied together throughout the entire network.
2. We examined the effect of intersegmental variability on the generation of locomotor output. For each simulation, the excitatory and inhibitory connection strengths *b* and *d* between different segments were sampled from a Gaussian distribution with a given mean and variance.

**Figure 5:**
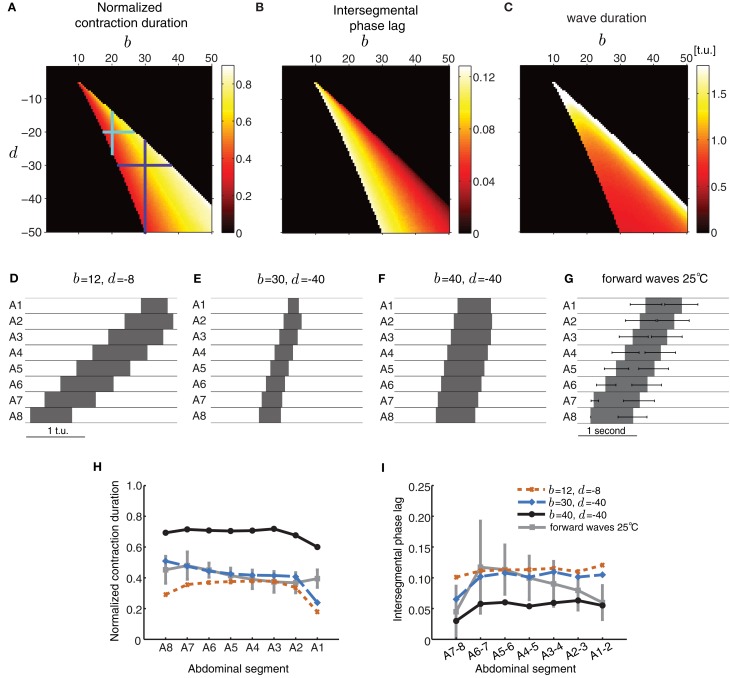
**Excitatory and inhibitory connectivity strength modulates wave properties in the CPG model. (A–C)** Effects of varying connection strengths upon the normalized contraction duration **(A)**, intersegmental phase lag **(B)**, and wave duration **(C)**. Value of 0 indicates that no wave was generated. For a given pair of (b,d) that produces waves, the total extent of variations in inhibition *d* that preserve wave generation (vertical line through the point (*b*, *d*)) is larger than the extent of variations in excitation *b* (horizontal line through the point (*b*, *d*)) as shown by the cyan and purple crosses. **(D–F)** Forward waves generated by the network with parameters specified in each panel. For all panels, the other parameters were set as in Table 1 and *P*_ext_ was applied to *E*_8_ for a sufficient duration to initiate a single forward wave. **(G)** Summary of the forward waves at 25°C from Figure 1B. **(H,I)** The normalized contraction duration **(H)** and intersegmental phase lag **(I)** for the three example waves in **(D–F)**. For comparison, we have also plotted the experimental data from forward waves at 25°C (Figures 1D,E).

In the first case, we chose a particular value for *b* and *d*, and examined segment activity duration and timing properties during wave propagation (Figure 5). Strengthening the excitatory connections (*b*) more than the inhibitory connections (*d*) increased the normalized segment activity duration (Figure 5A) and decreased the intersegmental phase lag (Figure 5B) consistent with increasing excitation or decreasing inhibition. A strong imbalance between excitatory and inhibitory connection strength completely eliminated waves. Figures 5A–C shows several parameter combinations which allow the model to generate waves. These waves have distinct properties depending on the specific value of connectivity pairs (b,d) (Figures 5D–F). The waves in **(D** and **E)** have similar normalized segment activity duration and intersegmental phase lag but different absolute speed of propagation (Figures 5H,I). The wave in panel **(F)** has different propagation properties: its segments are suprathreshold for a significant proportion of the wave duration and the wave propagates faster, i.e., intersegmental lags are shorter (Figures 5H,I). For comparison, we have also shown a summary of the forward waves at 25°C in Figure 5G.

Figures 5A–C also shows that wave generation in the model is more robust to changes in the inhibitory connectivity, *d*, than changes in the excitatory connectivity, *b*. For a given pair of (*b*, *d*) that produces waves, the total extent of variations in inhibition *d* that preserve wave generation (vertical line through the point (*b*, *d*)) is larger than the extent of variations in excitation *b* (horizontal line through the point (*b*, *d*)) (e.g., cyan and purple crosses in Figure 5A).

**Figure 6:**
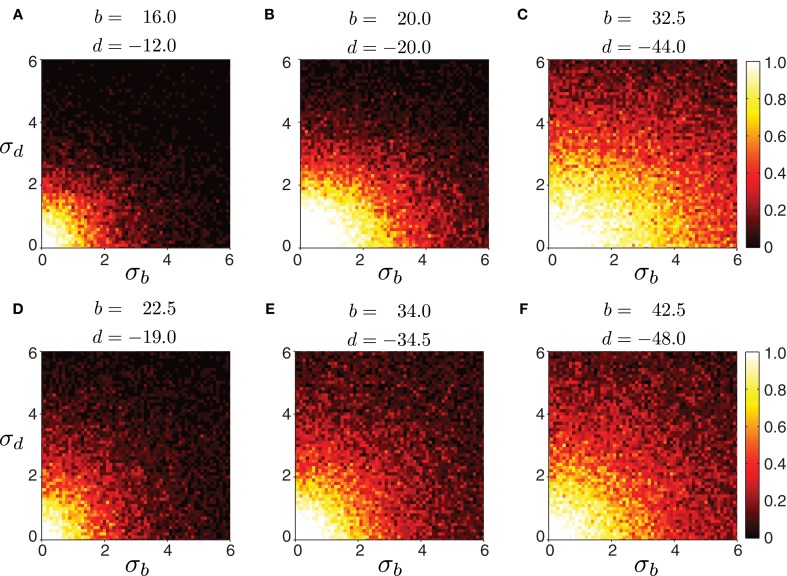
**Robustness of forward peristaltic waves generated by the CPG network model as excitatory and inhibitory connection strengths between segments are sampled from a Gaussian distribution.** The colorbar indicates the fraction of trials (20 trials per panel) when the model generated waves. **(A–F)** Connections were sampled from a Gaussian distribution with means *b* and *d* given in each panel, and standard deviation σ_*b*_ and σ_*d*_ varying as shown on each axes (weights were not allowed to change sign even when the noise level was high). Remaining parameters were set as in Table 1. The particular intersegmental connectivity values (*b* and *d*) were chosen such that the waves in **(A–C)** had average normalized segment duration of 0.42, while the waves in **(D–F)** of 0.57.

Biological systems, however, are inherently variable. Therefore, we tested the robustness of the model to variability in connection strength. We started with a network whose excitatory and inhibitory connection strengths were such that waves could be generated with appropriate timing properties. Then, we perturbed connections by adding noise sampled from a Gaussian distribution with a mean fixed to the chosen values, and a given variance. We examined whether wave generation was still possible as the variability in connection strength increased. Figure 6 shows the fraction of propagating waves from 20 simulations for a given set of initial (*b*, *d*) parameters. The model is reasonably robust to noise: larger mean values of *b* and *d* increase robustness. Therefore, strongly connected networks are more likely to generate waves even if connection strength is not precisely tuned. In contrast, weakly connected networks require more precise tuning of excitation and inhibition to generate appropriately-timed propagating waves.

### Sensory feedback improves wave propagation

Stretch-sensitive sensory neurons have been demonstrated to strongly affect the pattern of wave propagation (Hughes and Thomas, 2007; Song et al., 2007). Two classes of multiple-branched (*md*) sensory neurons provide the majority of feedback during normal larval crawling: the bipolar dendritic (*bd*) neurons, and the class I dendritic arborization (*da*) neurons (Hughes and Thomas, 2007). The dendrites of both classes of neurons are attached to the epidermis innervating a receptive area, which spans the width of each segment (Grueber et al., 2002; Schrader and Merritt, 2007). They become activated when tension increases in neighboring segments, providing feedback about the propagating wave (Merritt and Whitington, 1995; Hughes and Thomas, 2007; Simon and Trimmer, 2009). We therefore examined how such sensory feedback could be integrated into our model to adequately modulate wave propagation.

**Figure 7:**
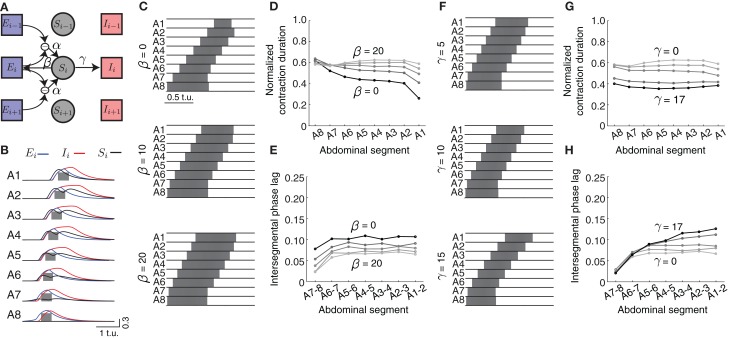
**Effects of sensory feedback on wave propagation in the CPG network model. (A)** Schematic of the model with sensory feedback. The activity in sensory neuronal populations (*S*_*i*_ for *i* = 1, …, 8) increases as a function of the difference in excitatory activity between neighboring segments through the connection α. This activity provides additional excitatory input to both excitatory and inhibitory populations in each segment, with connections denoted with β and γ, respectively. (Connections are shown for only one segment *i*). **(B)** The excitatory (blue), inhibitory (red), and sensory activity (black) for the eight segments during a forward wave as a function of time (here α = 25, β = 20, and γ = 17). We also show suprathreshold excitatory activity for each segment (gray). **(C)** The suprathreshold excitatory activity as excitatory connection β from the sensory population to the excitatory population increases. Parameters: α = 25, β ∈ {0, 10, 20}, γ = 0, θ_*C*_ = 0.3. **(D,E)** Normalized segment contraction duration and intersegmental phase lag for waves in **(C)** and β ∈ {0, 5, 10, 15, 20}. **(F)** The suprathreshold excitatory activity as excitatory connection γ from the sensory population to the inhibitory population increases. Parameters: α = 25, β = 20, γ ∈ {5, 10, 15}, θ_*C*_ = 0.3. *P*_ext_ was applied to *E*_8_ for 2.5 t.u. to initiate a single forward wave. Other parameters were as in Table 1. **(G,H)** Normalized segment contraction duration and intersegmental phase lag for the waves in **(F)** and γ ∈ {0, 5, 10, 15, 17}.

We introduced a “sensory” population that becomes activated by differences in excitatory activity between neighboring segments (Figure 7A, modulated by α in Equations 8–10) mimicking stretch sensing. We connected the sensory populations to both excitatory and inhibitory populations in the absence of more detailed connectivity information (Figure 7A, parameters β and γ, respectively). Figure 7B shows the activities of all neuronal populations in the model during forward wave propagation. Sensory activity in the middle segments exhibited two peaks, because of the “stretch” coming from the excitatory activity in each of the two neighboring segments. Also, in more anterior segments, sensory activity occurred before excitatory activity, indicative of its effect on promoting forward propagation.

We modulated the different strengths of excitatory (β) and inhibitory (γ) connections from the sensory population (Figures 7C,F) to examine how they might affect the timing relationships between segments. As the excitation provided by β increased (keeping γ = 0), the excitatory population in segments A1–A7 was suprathreshold progressively longer (Figure 7D). This additional excitation allowed for each segment to be suprathreshold for a similar time (compare to Figure 3B). However, in general all segments were suprathreshold for longer (~0.6 of the wave duration) than observed in the experimental data (Figure 1). To achieve the observed duration of ~0.41, we increased the inhibition strength γ (Figure 7G).

The intersegmental phase lag decreased equally for all segment pairs when the excitation strength from the sensory population β increased, thus preserving the same phase relationship between segment pairs as without sensory input (Figure 7E). Smaller intersegmental phase lag implies that activity propagates faster from one segment to another, which is expected given the increased input into the excitatory population from the sensory neurons. Increasing inhibition strength from the sensory population (γ) increased the intersegmental phase lag for the anterior, but not posterior, pairs of segments (Figure 7H). In summary, additional excitatory drive coming from the sensory population increases the speed of wave propagation and generates a more uniform pattern of segment contraction duration in the CPG network.

**Figure 8:**
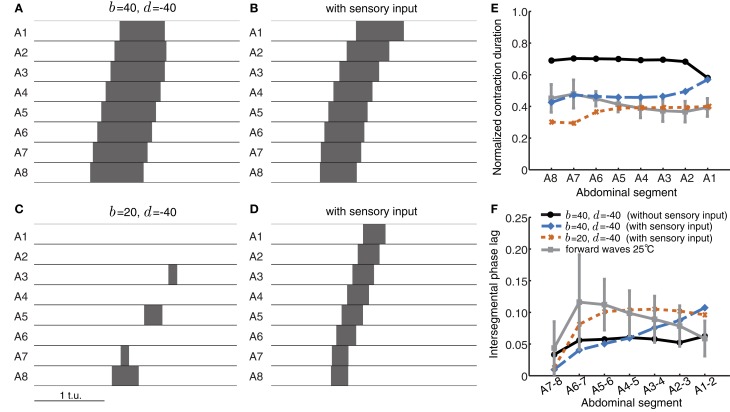
**Sensory feedback alters timing properties of wave propagation in the CPG network model. (A)** The wave from Figure 5F without any sensory feedback. **(B)** The wave in **(A)** with sensory feedback parameters α = 25, β = 45, and γ = 55. **(C)** Weak excitatory connections prevent wave initiation. **(D)** As **(C)** but with sensory feedback (α = 25, β = 40, γ = 0). The additional excitation from the sensory population rescues wave propagation, bringing excitatory activity above threshold. *P*_ext_ was applied to *E*_8_ for a duration of 0.8 t.u. in **(A,B)** and 1.2 t.u. in **(C,D)** to initiate a single forward wave. Other parameters were as in Table 1. **(E,F)** Normalized segment contraction duration and intersegmental phase lag for the waves in (**A,B,** and **D**) (no waves generated for **C**). For comparison, we also show the experimental data from forward waves at 25°C (Figures 1D,E).

Sensory feedback mechanisms could also improve the reliability of wave propagation against perturbations and noise. We therefore evaluated whether sensory feedback could rescue wave propagation in conditions when intersegmental connectivity was inappropriately tuned. Figure 8A shows an example in which although segmental activity crossed threshold in the correct order, suprathreshold activity rapidly spread to neighboring segments and stayed above threshold for a long time. Thus, the wave had longer normalized suprathreshold activity than the experimentally-observed value of 0.41 (Figure 8E) and shorter intersegmental phase lag than the experimentally-observed value of ~0.09 (Figure 8F). Adding sensory feedback brought wave properties closer to the experimentally-observed values (Figures 8B,E,F). Sensory feedback also rescued wave propagation in a case where inhibition was too strong to generate waves (Figure 8C). An example of a wave restored by sensory feedback is shown in Figure 8D and quantified in Figures 8E,F.

In summary, we have shown that adding sensory feedback significantly modulates wave propagation patterns in the model. Although CPG networks can generate propagating waves on their own, sensory feedback brings segmental contraction duration and speed of propagation closer to experimental observations. This sensory feedback also rescued the timing relationships during wave propagation in networks where excitation and inhibition were inappropriately tuned.

### A two-sided model can generate synchronous activity

Drosophila larvae crawl in circles when the left and right side contractions differ in magnitude; they can also turn by producing a unilateral backward peristaltic wave in the most anterior segments (Berni et al., 2012), suggesting the existence of bilaterally located CPGs. Left and right hemisegments contain identical neurons which need to be synchronized to allow the larvae to crawl in a coordinated fashion. Thus, we sought to find the types of connectivity across the midline that could synchronize activity in corresponding segments of two independent networks. For computational tractability, we considered only short-range connectivity between neuronal populations within the same segment.

**Figure 9:**
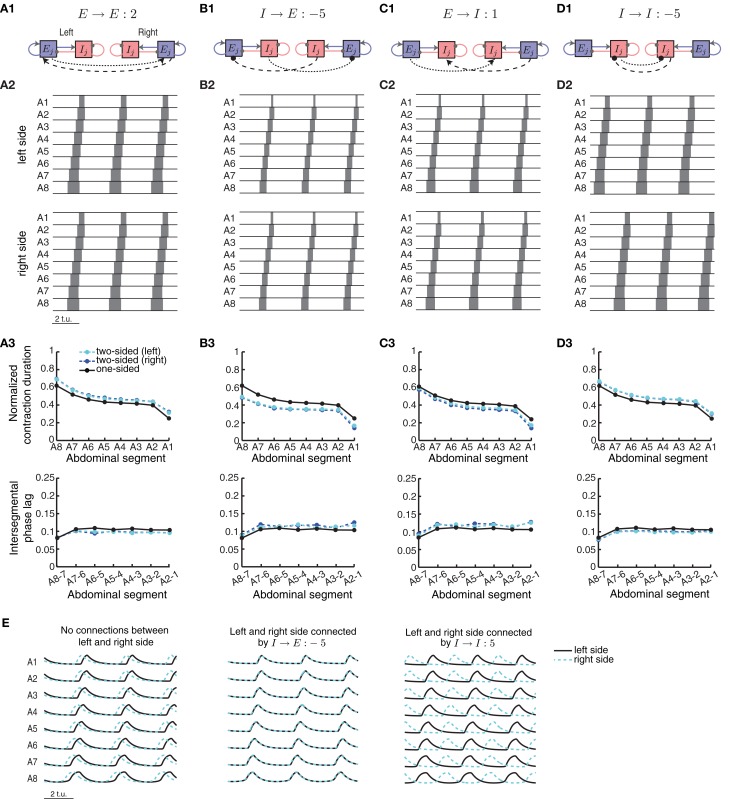
**A two-sided CPG network model for synchronous wave propagation.** The CPG network model for wave propagation in Figure 2 (without sensory feedback) was replicated for each side of the body to model activity in the two sides of the *Drosophila* larval body. Four types of connections within each segment were evaluated for synchronizing activity in the two sides: **(A1)** excitatory–excitatory, **(B1)** inhibitory– excitatory, **(C1)** excitatory–inhibitory, and **(D1)** inhibitory–inhibitory. Only one segment (*j*) is shown, but all segments are identically coupled. **(A2,B2,C2,D2)** Typical left and right suprathreshold excitatory activity in the two-sided model for one example of each of the four possible types of contralateral connections. In each case we used a threshold of θ_*C*_ = 0.3 for detecting suprathreshold activity. The values of the contralateral connections were chosen so that a synchronous wave was generated, if possible, based on Figure 10. **(A3,B3,C3,D3)** Normalized segment contraction duration and intersegmental phase lag for the waves in **(A2,B2,C2,D3)**; also compared to the one-sided model with the same intersegmental connection parameters. **(E)** (Left) *P*_ext_ = 1.7 was applied to *E*_8_ on the left side and *P*_ext_ = 1.72 on the right side of the network, for a duration of 35 t.u. In the case of no connectivity between the two sides, the waves eventually de-synchronized despite the small difference in external input on each side. (Middle) Left/right *synchronous* activity for one example of the *I* → *E* connection **(B2)**. (Right) Left/right *asynchronous* activity for one example of the *I* → *I* connection **(D2)**. In all cases we show the last 9 t.u. of the simulated waves.

Bifurcation analysis of a much simpler system consisting of two coupled EI units highlighted conditions for synchronization of the two units with four different types of connections (Borisyuk et al., 1995). We examined the same four types of connections in a two-sided model of eight segments, shown in Figure 9: **(A)** connections between the two excitatory populations on the left and right sides of the same segment, *E* → *E*, **(B)** connections from the inhibitory population on one side to the contralateral excitatory population, *I* → *E*, **(C)** connections from the excitatory population on one side to the contralateral inhibitory population, *E* → *I*, and **(D)** connections between the two inhibitory populations on the left and right sides, *I* → *I*. All these connections may be present and acting simultaneously, however, we analyze how they independently affect network dynamics to understand which connectivity patterns might be necessary to synchronize the two networks.

**Figure 10:**
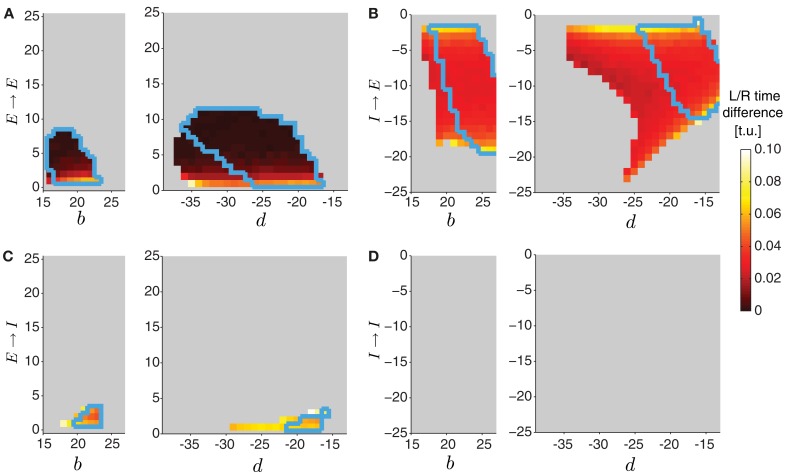
**Varying the connectivity between the two sides in the two-sided model.** In the two-sided model in Figure 9, we varied one of the intersegmental connections *b* or *d* together with one of the four contralateral connection types (*E* → *E* **(A)**, *I* → *E* **(B)**, *E* → *I* **(C)**, *I* → *I* **(D)**). The other intersegmental connection was set to a particular value, *b* = 20 if *d* varied, or *d* = −20 if *b* varied. The color indicates the time difference (after disregarding transient activity) between threshold crossings of excitatory activity on the left and the right side (averaged across segments). Time differences above 1 t.u. were considered asynchronous, and were not analyzed (colored gray). In all cases we quantified waves using threshold for detecting suprathreshold activity θ_*C*_ = 0.3 (colored regions inside the blue boundaries) and θ_*C*_ = 0.2 (entire colored regions).

To start a wave in this two-sided model, we applied external drive to the most posterior excitatory unit *E*_8_ in each, left and right, network for forward propagation. Each network, however, received a slightly different external drive such that even though wave properties were identical, activity was misaligned (Figure 9E, left). External drive was applied for a sufficient duration (35 t.u.) and waves were analyzed after eliminating transient dynamics, thus we observed multiple waves consistent with Figure 4. We examined how changing the strength of the four types of contralateral connections affected wave propagation and synchronization between the two networks. Because these connections modify the relative extent of excitation and inhibition received by each segment, investigating the effects of these contralateral connections was done concurrently with the intersegmental connections on each side, *b* and *d*. In Figure 10A we show the average time difference between segmental activation of the left and right network for a different pair of connection strengths (*b*, *E* → *E*) (left) and (*d*, *E* → *E*) (right). The figure shows that segmental activity in the left and right network is synchronous when the *E* → *E* connection takes values less than 10. When the total amount of inhibition received by a segment increased with *d*, the activity of each segment was below 0.3, so we used threshold of 0.2 to quantify the resulting waves. Using this lower threshold for the detection of suprathreshold activity produced left/right synchronous waves over a larger range of parameters (blue boundary in Figure 10A denotes the 0.3 threshold).

One example of synchronous wave activity generated by our two-sided model with *E* → *E* connection of strength 2 is shown in Figure 9A2. Figure 9A3 shows that the normalized contraction duration in the two-sided model was slightly longer than for the one-sided model with the same connection strengths. This was due to increased excitation in the model coming from the *E* → *E* connection. The effect was amplified for even stronger *E* → *E* connections, eventually saturating excitatory activity and eliminating waves when the connection strength *E* → *E* exceeded approximately 10 (Figure 10A). The intersegmental phase lag for the activity of each side in the two-sided model was similar to the one-sided model (Figure 9A3).

The model with *I* → *E* connections generated synchronous waves over the largest range of parameters compared to the other types of contralateral connections (Figure 10B). Because the total inhibition increased with stronger *d* or *I*→ *E*, the overall activity levels were lower. Using threshold of 0.2 increased the range of parameters over which left/right synchronous waves were observed (blue boundary in Figure 10B denotes the 0.3 threshold). Connecting the left and the right side with *I* → *E* equal to −5 (Figure 9B2) synchronized the two networks without affecting the timing relationships during wave propagation (Figure 9E, middle). The normalized contraction duration and the intersegmental phase lags are shown in Figure 9B3 for the left and the right waves, and are compared to a one-sided wave generated with the same connectivity strengths.

The two-sided model with the *I* → *I* connection always produced alternating left/right activity (Figures 9D2 and E, right). Therefore, Figure 10D shows that synchronous waves were produced for no range of parameters. The normalized contraction duration and the intersegmental phase lags are shown in Figure 9D3 for the left and the right waves, and also for a wave generated with the one-sided model using the same connectivity strengths. After further increasing the *I* → *I* connection strength, the excitatory activity on each side became independent, and the normalized contraction duration for each side was the same as in the one-sided model (data not shown). Alternating activity was also observed in the majority of the models with *E* → *I* connections, except for a small range of parameters for which the excitatory activity of the left and the right sides was synchronized (Figure 10C). We show one wave in Figure 9C2 with the quantification in Figure 9C3. Very strong connections completely eliminated the excitatory activity on one side of the model, while preserving the waves on the other side. When comparing the two-sided to the one-sided wave properties, we observed that increasing excitation (*E* → *E*) or decreasing inhibition (*I* → *I*) increased segment contraction duration in the two-sided model waves, while increasing inhibition (*E* → *I*) or decreasing excitation (*I* → *E*) decreased segment contraction duration in the two sided-model waves. Consequently, in the former case the two-sided waves propagated faster, while in the latter case they propagated slower than the one-sided model waves.

This work suggests that a two-sided model which generates synchronous left/right side excitatory activity can be constructed by simply connecting two networks, each representing a one-sided CPG model. We found that, in general, only contralateral connections into the excitatory neuronal populations (*E* → *E* and *I* → *E*) synchronized left and right activity in the network, but for a limited strength of this connectivity. The other two connection types produced left/right alternating activity for all (*I* → *I*), or for the majority of connection strengths (*E* → *I*) in our model.

## Discussion

The rhythmic behavior of many organisms can be described by the activity of a CPG (Grillner, 2003, 2006; Marder et al., 2005; Feldman and Del Negro, 2006). In most locomotor circuits (lamprey, *Xenopus*, leech), coordinated motor patterns are produced by left-right (dorso-ventral in the case of leech) alternating activity of two reciprocally-coupled half center oscillators (Grillner, 1985, 2003; Kopell and Ermentrout, 1988; Tunstall et al., 2002; Hill et al., 2003; Roberts et al., 2010). Unlike these CPG circuits, rhythm generation in the nematode *C. elegans* is governed by sensory feedback within the motor circuit; yet locomotion consists of an alternating pattern of dorso-ventral contractions which propagate from head to tail generating a sinusoidal wave pattern of muscle contractions along the worm body (Wen et al., 2012). Such models fail to describe the synchronous propagation of muscle contractions on both sides of the midline during crawling in *Drosophila*. Furthermore, lamprey and *Xenopus* do not have clearly defined segments along the body axis, but consist of many (possibly hundreds) repeated units or network elements that produce a quasi-sinusoidal wave that travels along the body with a constant phase shift (Kopell and Ermentrout, 1988; Zhaoping et al., 2004; Grillner, 2006). In contrast, the *Drosophila* larval body consists of only eight segmentally repeated units which contract sequentially during a propagating wave in a state-dependent manner (Crisp et al., 2008; Dixit et al., 2008; Lahiri et al., 2011; Berni et al., 2012).

Models for leech swimming (Cang and Friesen, 2002) based on the experimentally identified architecture (Friesen, 1989a,b; Brodfuehrer et al., 1995) can generate the constant phase lags observed during swimming, suggesting that some of the network motifs responsible for the generation of coordinated motor output might be shared among species. CPG models for crawling in leech and caterpillar have also been developed (Cacciatore et al., 2000; Trimmer et al., 2006). Leech crawling consists of alternating elongation and contraction patterns (Eisenhart et al., 2000; Friesen and Kristan, 2007; Puhl and Mesce, 2008), and in addition uses descending inputs that ensure intersegmental coordination of the locomotion oscillators (Puhl and Mesce, 2010). However, the waves of activity during swimming and crawling in leech always progress along the body from anterior to posterior end (Friesen and Cang, 2001; Puhl and Mesce, 2008) unlike *Drosophila*. Furthermore, leeches also cannot swim backwards, which is supported by the asymmetric intersegmental connectivity along the body (Cang and Friesen, 2002). All of these reasons suggested to us that central pattern generation in *Drosophila* crawling is different from other locomotor behaviors previously studied.

Despite the wealth of knowledge about the neuronal identity and morphology of local and intersegmental interneurons in *Drosophila*, few studies have investigated the neuronal composition of the crawling CPG. It is known that the circuitry for crawling is located in segments posterior to the suboesophageal ganglion and that it must contain numerous interconnected interneurons, but no detailed information exists on the neuronal identity of the CPG components (Suster et al., 2003; Iyengar et al., 2011; Berni et al., 2012). For this reason, we proposed a model based on a coupled neuronal populations. Each segment was modeled as a single unit consisting of an excitatory and an inhibitory population (Wilson and Cowan, 1972). Models of neuronal populations have been previously used even for systems where the neural properties are known such as the lamprey CPG (Zhaoping et al., 2004; Grillner, 2006; Mullins et al., 2011). Similar mean field models have also been used to explain differences in episodic activity generated spontaneously in the developing spinal cord by representing only average activity rather than individual spikes times (Vladimirski et al., 2008; Tabak et al., 2010). Therefore, the neuronal population-based framework we have implemented is a good starting model for the central networks involved in *Drosophila* larval crawling. Our model can be further refined when distinct central neuronal populations in *Drosophila* become characterized in the future.

We examined how well our proposed model captured the propagating peristaltic waves anteriorly along the body axis during larval crawling (Crisp et al., 2008; Dixit et al., 2008; Lahiri et al., 2011; Berni et al., 2012; Heckscher et al., 2012). We quantified such locomotor patterns by recording the movement of segment boundaries during peristaltic crawling. These boundaries move as a consequence of muscle contractions and they are the basic unit for peristaltic locomotion at the body wall level (Lahiri et al., 2011; Berni et al., 2012). Even though the dynamics of contraction of particular muscles have been described (Heckscher et al., 2012), our premise is that such biophysical realism at the muscular level is not critical for capturing the properties of the waves generates by circuits in the parasegmentally organized central nervous system (Landgraf et al., 2003). We reported the duration of contraction for each segment and the intersegmental phase lag between neighboring segments (in agreement with Heckscher et al. (2012)). We first tested the basic requirement for the propagation of neuronal activity (without sensory feedback) consistent with crawling in *Drosophila* larvae [Figure 1; Heckscher et al. (2012)]. We used units of excitatory and inhibitory populations to represent each segment in the network, and determined how these must couple to neighboring segments to support wave propagation. We found that short range connectivity, spanning nearest-neighbor segments only, was sufficient to generate waves which propagate with properties similar to experimental data. To what degree this is consistent with intersegmental connectivity in *Drosophila* larvae needs to be addressed. For example, the chain of abdominal segments could be shortened to a pair of segments with optogenetic tools or surgical ablation to test whether propagating muscle contractions can still be produced in the complete absence of long-range connectivity.

The requirement for short range connectivity for normal propagation of peristaltic waves is consistent with experimental evidence that the CPG for crawling in *Drosophila* is locally distributed. In that context, Dixit et al. (2008) have shown that homeotic transformation of three thoracic segments into abdominal identity is sufficient to incorporate them as part of the peristaltic wave. However, this is not the case in other animals where CPGs underlie locomotion. For instance, intersegmental coupling in leech spans approximately six segments (Poon et al., 1978; Friesen and Hocker, 2001), although modeling has shown that intersegmental interactions must be relatively weak compared to oscillator interactions within intersegmental ganglia (Zheng et al., 2007).

Moderately modulating levels of excitation and inhibition in our network model did not disrupt wave propagation. Waves, however, were more robust to changes in inhibition than excitation. This may suggest that the strength of inhibitory input is less precisely regulated than that of excitatory inputs, which in turn, could result in higher variability of the patterns of inhibitory connectivity. This may also suggest possible differences between excitatory and inhibitory synaptic plasticity mechanisms acting during development, for instance, excitatory and inhibitory connection strengths may be tuned following different spike timing rules. Very little is known about inhibitory plasticity and only in vertebrate neural circuits [reviewed in Kullmann et al. (2012)]; most studies have focused on the plasticity of excitatory synapses (Bi and Poo, 1998; Sjöström et al., 2001). Although Hebbian style plasticity was characterized in the olfactory system in insects (Cassenaer and Laurent, 2007) it is unclear what the implications are for the tuning of excitation and inhibition in a motor network. Such questions could be investigated by selectively blocking excitatory or inhibitory activity during development when activity-dependent mechanisms appear to play an important role for the appropriate tuning of network connectivity (Crisp et al., 2008, 2011).

In our model strongly-connected networks were more likely to generate waves even if connection strength was not precisely tuned. In contrast, weakly connected networks required precise tuning of excitation and inhibition to generate appropriately-timed propagating waves. Although this would always argue for strongly-connected networks, there is most likely a trade-off between robust networks and the cost imposed by metabolic constraints of maintaining strong synapses (Chklovskii, 2000; Attwell and Laughlin, 2001; Wen and Chklovskii, 2008). It is therefore likely that there are many feasible solutions that can generate coordinated network activity as has been shown in other experimental systems (Marder, 2011).

We have based our model on the assumption that rhythmic output during crawling in *Drosophila* larvae is generated by interconnected CPGs in the absence of any sensory feedback. This assumption is supported by many experiments which have blocked sensory input in the embryo and the larva and observed that rhythmic output is still generated, albeit with altered wave properties (Suster and Bate, 2002; Hughes and Thomas, 2007; Crisp et al., 2008; Berni et al., 2012) [although see Song et al. (2007)]. Therefore, our model differs from models of other systems (for instance, *C. elegans*) where sensory input is a critical element of the pattern generating mechanisms (Bryden and Cohen, 2008).

Behavioral experiments in freely moving *Drosophila* larvae have shown that sensory feedback affects the speed of wave propagation leading to an abnormal pattern of muscle contraction (Suster and Bate, 2002; Hughes and Thomas, 2007). However, these experiments lacked a detailed evaluation of the phase relationship between contractions of neighboring segments and the speed of wave propagation. Our modeling results showed that adding sensory feedback to our CPG model affected both speed and intersegmental phase relationship. Both experimental and modeling results from swimming circuits in leech have demonstrated that sensory feedback alters the phase of the local central oscillators producing the rhythm (Cang and Friesen, 2000, 2002). Such sensory input does not appear necessary in the swimmeret system of crayfish or for swimming in adult lamprey (Friesen and Cang, 2001). It is likely that such species differences have evolved due to the different environments to which each species has adapted. It will be interesting to compare these differences with *Drosophila* as an experimental system, and to our modeling predictions. To our knowledge, it is not known how sensory feedback connects to the CPG network in *Drosophila*. We have varied the sensory input to both inhibitory and excitatory neuronal populations and demonstrated that intersegmental phase lag is predominantly affected by connecting the sensory input to the inhibitory population, whereas connecting sensory input to the excitatory population primarily regulates the contraction duration.

When CPG connectivity was poorly tuned in our model such that the network failed to generate propagating waves, we showed that adding sensory feedback rescued wave propagation. Patterned sensory feedback is not required for the initial development of coordinated peristalsis (Suster and Bate, 2002). Crisp et al. (2008) have proposed that network connectivity undergoes a period of refinement following the first generation of peristaltic waves. It would be informative to test whether sensory input might influence the successful maturation of crawling by acutely removing sensory input at late stages of embryogenesis. Our model predicts that embryos raised with normal sensory feedback should produce propagating waves more robustly even when CPGs have not yet fully matured.

We have proposed connectivity patterns that can support synchronization of contraction between right and left side of the body. In particular, connection types which increased the amount of excitation (*E* → *E*) succeeded in achieving network synchronization. Consistent with Borisyuk et al. (1995), we found that synchrony was produced for only small values of connection strength. Synchronization with commissural excitatory connections has been observed in the CPG for breathing (Feldman and Smith, 1989; Shao and Feldman, 1997; Feldman and Del Negro, 2006), however, these systems exhibit only temporal rhythm generation, rather than longitudinal coordination of multiple segments as observed during crawling in *Drosophila* larvae. In rodent CPG for locomotion, excitatory glutamatergic commissural neurons that project directly to motor neurons are thought to be active during hopping, a condition of synchronous activity (Quinlan and Kiehn, 2007). The role for contralateral excitatory connections for synchronized gait is also supported by evidence that reducing the number of inhibitory connections across the midline synchronizes left and right side in mice and horse (Kullander et al., 2003; Restrepo et al., 2011; Andersson et al., 2012).

In our two sided model, commissural inhibitory connections connecting the inhibitory population on one side to the inhibitory population on the other side (*I* → *I*) always produced alternating left/right activity. Although this is consistent with modeling and electrophysiological data in lamprey and *Xenopus* tadpole (Soffe et al., 1984; Wang and Rinzel, 1992; Borisyuk et al., 1995; Kiehn, 2011), the inhibitory interneurons in these systems project contralaterally to both excitatory and inhibitory neurons, thus both types of *I* → *E* and *I* → *I* connections are simultaneously present, a condition which we did not consider in our model. A further difference in our model from these systems is the presence of the *I* → *E* connection *within* a segment; as part of the Wilson–Cowan oscillator for the model of each segment, this type of connection contributes to the generation of rhythmic behavior that we observed.

Interestingly, we showed that connections from inhibitory populations on one side to contralateral excitatory population (*I* → *E*) generated synchronous waves of activity over the largest range of parameters. Such type of commissural neurons have not been described so far experimentally, but they may well be present in the larva. Our prediction of the existence of this type of *I* → *E* connection was only possible because we model rhythm generation along the body as well as activity synchronization across the body. Borisyuk et al. (1995) also found that the *I* → *E* connection can lead to left/right synchrony in a model of a single segment. However, their observation was true only for weak connection strength; increasing the connection strength produced anti-phase oscillations, eventually leading to chaotic dynamics. These differences could be explained by the additional coupling that *E* and *I* populations in our eight segment model receive from neighboring segments. We focused on determining the conditions for synchronous left/right activity because of their relevance to *Drosophila* larval crawling; therefore, we did not systematically explore other oscillatory regimes for our model.

In summary, we have proposed a modeling framework which captures the neuronal dynamics essential for generating propagating peristaltic waves by a simple CPG architecture with a minimal number of parameters. Two other styles of modeling lie at each extreme of our chosen framework. On one end are phase coupled oscillator models which ignore the neuronal dynamics and have a limited ability to make predictions related to rhythm generation (Kopell and Ermentrout, 1988; Skinner and Mulloney, 1998a). On the other end are detailed biophysical models which incorporate detailed descriptions of known neurons and connections (Ekeberg et al., 1991; Skinner and Mulloney, 1998b). As detailed experimental data about the circuits involved in wave propagation in *Drosophila* are not yet available, we believe that these detailed biophysical models are not yet appropriate for our system. We provide an alternative framework of moderate complexity avoiding nonessential detailed dynamics and specific biophysical details (such as muscle dynamics). Through numerical simulations we determine relevant parameter ranges which achieve good fit of behavioral data and make several predictions. Therefore, our model demonstrates that the abstraction of biophysical phenomena into segmental units with the appropriate connections between them is sufficient to generate the observed rhythmic patterns.

### Conflict of interest statement

The authors declare that the research was conducted in the absence of any commercial or financial relationships that could be construed as a potential conflict of interest.
